# Using Forecast and Observed Weather Data to Assess Performance of Forecast Products in Identifying Heat Waves and Estimating Heat Wave Effects on Mortality

**DOI:** 10.1289/ehp.1306858

**Published:** 2014-05-09

**Authors:** Kai Zhang, Yeh-Hsin Chen, Joel D. Schwartz, Richard B. Rood, Marie S. O’Neill

**Affiliations:** 1 Division of Epidemiology, Human Genetics and Environmental Sciences, University of Texas School of Public Health, Houston, Texas, USA; 2 Department of Environmental Health Sciences, University of Michigan, Ann Arbor, Michigan, USA; 3 Department of Environmental Health, Harvard University, Boston, Massachusetts, USA; 4 Department of Atmospheric, Oceanic, and Space Science,; 5 Department of Epidemiology, and; 6 Risk Science Center, University of Michigan, Ann Arbor, Michigan, USA

## Abstract

Background: Heat wave and health warning systems are activated based on forecasts of health-threatening hot weather.

Objective: We estimated heat–mortality associations based on forecast and observed weather data in Detroit, Michigan, and compared the accuracy of forecast products for predicting heat waves.

Methods: We derived and compared apparent temperature (AT) and heat wave days (with heat waves defined as ≥ 2 days of daily mean AT ≥ 95th percentile of warm-season average) from weather observations and six different forecast products. We used Poisson regression with and without adjustment for ozone and/or PM_10_ (particulate matter with aerodynamic diameter ≤ 10 μm) to estimate and compare associations of daily all-cause mortality with observed and predicted AT and heat wave days.

Results: The 1-day-ahead forecast of a local operational product, Revised Digital Forecast, had about half the number of false positives compared with all other forecasts. On average, controlling for heat waves, days with observed AT = 25.3°C were associated with 3.5% higher mortality (95% CI: –1.6, 8.8%) than days with AT = 8.5°C. Observed heat wave days were associated with 6.2% higher mortality (95% CI: –0.4, 13.2%) than non–heat wave days. The accuracy of predictions varied, but associations between mortality and forecast heat generally tended to overestimate heat effects, whereas associations with forecast heat waves tended to underestimate heat wave effects, relative to associations based on observed weather metrics.

Conclusions: Our findings suggest that incorporating knowledge of local conditions may improve the accuracy of predictions used to activate heat wave and health warning systems.

Citation: Zhang K, Chen YH, Schwartz JD, Rood RB, O’Neill MS. 2014. Using forecast and observed weather data to assess performance of forecast products in identifying heat waves and estimating heat wave effects on mortality. Environ Health Perspect 122:912–918; http://dx.doi.org/10.1289/ehp.1306858

## Introduction

Heat waves have been linked to increased risk of mortality, hospital admissions, heat stroke, heat exhaustion, and cardiovascular and respiratory diseases (Anderson and Bell 2009; Gasparrini and Armstrong 2011; Kovats and Ebi 2006; Kovats and Hajat 2007). Heat wave and health warning systems (HHWSs) are preparedness plans designed to reduce heat-related adverse health effects, and can help raise awareness among populations vulnerable to extreme heat. HHWSs have been established to issue heat advisories to the public based on weather forecast data in many cities in the United States and elsewhere (Kovats and Ebi 2006). Several triggers for HHWS algorithms have been proposed and implemented, as reviewed by Zhang et al. (2012), including absolute or relative temperature thresholds, the heat index, physiologically based discomfort classifications, temperature–mortality relationships derived from epidemiologic analysis, and spatial synoptic classification. The latter two methods use local heat–mortality associations derived from observed death counts and historical weather observations and then predict mortality risks for the next few days by linking these developed heat–mortality associations with weather forecasts. These analyses treat weather forecasts exactly the same as weather measurements when they use forecasts to issue heat alerts. Recently, Henderson and Kosatsky (2012) defined 4 days during 2005–2009 as heat health emergencies, first by examining the coincidence of extreme temperature days and extreme mortality days using archived data from a coastal airport and an inland airport in the Vancouver metropolitan area, Canada, and then by evaluating the predictive ability of heat alerts based on forecast data with different lead times. Henderson and Kosatsky (2012) found that the accuracy of heat alerts predicted by forecasted temperatures varied with lead time and geographical areas compared with those predicted by observed temperatures.

We know that forecast data vary in quality for different weather parameters. For example, temperature usually has a more accurate forecast than dew point temperature (DPT) (Werth and Garrett 2011). Werth and Garrett (2011) compared 1 year of predictions from the Global Forecast System (GFS) (a global numerical forecast model system) to nearly 12,000 ground stations, and reported that typical root mean square errors (RMSEs) were 3°C for air temperature and 3.5°C for DPT.

This is an important question for the design of HHWS because the use of the forecast parameters with performance most comparable to the observed weather in association with mortality to trigger public health interventions would be preferred.

The objective of this study was to investigate how well forecast models reproduce heat waves seen in the observations from Detroit, Michigan, using one definition of heat wave and the impacts of weather forecast quality on heat–mortality associations. Previous studies on heat–mortality associations in Detroit have reported that hot weather is significantly associated with excess mortality in this city, and heat has a disproportionate burden on people who have diabetes, are less educated, or are black—a disparity that could be explained in part by unequal access to home air conditioning (O’Neill et al. 2003, 2005; Schwartz 2005).

To inform our analysis, we followed the suggestion of Gasparrini and Armstrong (2011) to classify the estimated effects of temperature on mortality into two parts: *a*) the “main effect,” defined as the estimated independent effect attributed to daily temperature, and *b*) the estimated heat wave effect associated with heat waves lasting for ≥ 2 days. Here we refer to the “main effect” and “heat wave effect” as “heat” and “heat wave” effects (or associations). We applied generalized additive models to multiple time series of daily all-cause mortality counts and weather observations or archived weather forecasts from six weather forecast products in Detroit. We then assessed how consistent associations between mortality and predicted heat and heat waves were with estimated effects of observed heat and heat waves based on the same models.

## Methods

*Data sources*. Mortality data. We obtained daily all-cause mortality data from the Detroit metropolitan area (Wayne, Oakland, and Macomb counties in Michigan) in the warm season (1 May to 30 September) from 2002 to 2006. Original death records were obtained from National Center for Health Statistics (NCHS) and aggregated into daily counts. *The International Classification of Diseases, Tenth Revision* (ICD-10), was used to classify causes of death for the study period (World Health Organization 1992). Daily total mortality excluded deaths from external causes (i.e., ICD-10 codes S through Z, which include injuries, poisonings, and traffic accidents, among others).

Observed weather data. Hourly weather observations at the Detroit Metropolitan Airport monitoring station (station name: Detroit/Metropolitan) were obtained from the National Climate Data Center (NCDC 2010) in the study period. We then calculated maximum and minimum temperatures, dew point temperature, and apparent temperatures (AT) for each day in the time period and averaged the maximum and minimum ATs on each day to derive a daily average AT measure. ATs were derived using the standard formula previously reported (Zanobetti and Schwartz 2008).

We chose to use AT in this study because AT includes temperature and humidity information in a way similar to the heat index, and it is more easily applied than the heat index [which is limited to certain temperature thresholds (i.e., > 26.7°C) and relative humidity thresholds (i.e., > 40%)].

Forecast weather data. Weather forecast products are generated by postprocessing forecast output from several numerical weather prediction models using statistical approaches or local meteorologists’ judgments.

Model Output Statistics (MOS) products are generated by the National Weather Service’s (NWS) Meteorological Development Laboratory (NWS 2011). MOS products are “operational products,” that is, data sets used by forecasters in local NWS offices in the decision-making process to provide real-time weather forecasts. The MOS products are calibrated forecast outputs to bridge the gap between the original outputs of numerical forecast models and the observations in the NWS monitoring network (NWS 2011). This calibration is usually implemented by building multiple linear regression models that are based on historical weather observations and forecast outputs. Thus, the MOS products provide forecasts of weather variables by combining physically based numerical models and statistical models. MOS is also used to downscale weather predictions at a grid to local stations. Three major types of MOS products exist: the GFS, the Nested Grid Model (NGM), and North American Mesoscale model (NAM, formerly called the Eta model). GFS is a global numerical forecast model system operated by the National Centers for Environmental Prediction (NCEP) of the National Oceanic and Atmospheric Administration (NOAA) (NCEP 2007). It produces forecasts every 6 hr at horizontal grid lengths ranging from 35 km to 70 km. NGM is a numerical model run by NCEP, which produces forecasts twice per day. It uses an 80-km resolution grid over North America and a 160-km resolution grid over the oceans; however, NGM forecasts are no longer produced. NAM is a regional numerical weather prediction model and generates forecasts every 6 hr/day at a 12-km resolution.

The Revised Digital Forecast (RDF) data are an operational forecast product produced by local meteorologists according to the outputs of numerical weather models and MOS models, their judgments, in addition to other information such as weather soundings (R. Pollman, personal communication). Local meteorologists usually make a decision regarding issuing a heat alert by considering many factors, including predictions from numerical forecast models, MOS products, and other forecast products as well as their local knowledge and others (Pollman R, personal communication).

Six different weather forecast products were obtained for the 2002–2006 study period. We first obtained five MOS products to represent these three model types and short-range/long-range forecasts, namely: the GFS-based short-range MOS forecast product (MAV: 6–72 hr in advance for most weather parameters), GFS-based extended-range MOS forecast product (MEX: extended range, 24 and 192 hr), and GFS-based ensemble MOS forecast product (ENS), NGM-based MOS forecast product (FWC), and NAM-based MOS forecast product (MET). In addition, we had access to the RDF product and extracted one archived local forecast data set (station name: KDTX, Detroit/White Lake, MI) from RDF retained by the NCDC (2011). Forecast products include forecasts of three durations: 1-, 2-, and 3-day forecasts. Thus, we had 18 predictions on a given day (predictions of three different durations multiplied by six forecast products).

Air pollution data. Increases in daily air pollution concentrations have been associated with increases in mortality, and air pollution levels often covary with weather conditions. Therefore, we wanted to include air pollution concentrations in the models. Daily concentrations of ozone (O_3_) and particulate matter with aerodynamic diameter ≤ 10 μm (PM_10_) were obtained from the U.S. Environmental Protection Agency’s Aerometric Information Retrieval System (AIRS) monitoring network (http://www.epa.gov/ttn/airs/airsaqs/). Because the number and location of operating monitors can vary from day to day, daily mean concentrations in the Detroit metropolitan area were derived employing an algorithm previously used in air pollution epidemiological studies (Schwartz and Zanobetti 2000).

Preparation of the combined data set for analysis. We merged mortality data, weather observations, forecasts, and air pollution data by date. For both observed and forecast data, we defined a heat wave indicator as periods of ≥ 2 consecutive days with daily mean AT ≥ 95th percentile of the observed or predicted summertime distribution (1 May–30 September) determined separately for each year.

We used SAS (version 9.2; SAS Institute Inc., Cary, NC) to extract daily data from forecasts and observations and to calculate biases (predictions minus observed values) on a daily basis and summarized biases using average root mean squared errors.

*Statistical approach*. We quantified forecast product performance in identifying heat waves by calculating “false-positive” and “false-negative” days. False-positive days were days when a forecast product predicted a heat wave day that did not occur (based on the observed data). False-negative days were days when a forecast product failed to predict a heat wave day that did occur.

We employed generalized additive models (GAMs) to model mortality counts as a function of the continuous AT metrics and indicator variables representing heat waves. This was done across weather observations and forecasts in the warm-season (1 May–30 September) study period. GAMs are commonly used in air quality and air pollution/heat epidemiology studies where seasonal patterns in outcome variables (e.g., mortality) and nonlinear associations between health outcomes and, say, temperature, require additional modeling flexibility (Anderson and Bell 2009; Gasparrini and Armstrong 2011; Schwartz and Zanobetti 2000; Zhang and Batterman 2010). GAMs have the ability to characterize nonlinear relationhips between an independent variable and a dependent variable using parametric and nonparametric smoothing functions (Hastie and Tibshirani 1990).

Separate GAMs were fit to estimate associations between daily mortality counts (the outcome variable) and daily average AT metrics derived from weather observations and from the different forecast products with three lag periods. We simultaneously included daily AT (as a continuous variable), to estimate the effect of heat, and an indicator variable for heat wave days (HW*_t_* = 1 if day *t* was classified as part of a heat wave, 0 otherwise), to estimate the effect of heat waves. We assumed daily death counts followed an overdispersed Poisson distribution and modeled them as

Log [*E*(*Y_t_*)] = α + β *DOW_t_* + γ*YEAR_t_* + *S*(*AT_t_*_,_*_t_*_–1_) + *S*(*T_t_*) + ηHW*_t_*, [1]

where *E*(*Y_t_*) is the expected daily mortality counts on day *t*; α is the intercept; *DOW_t_* is a set of indicator variables for day of the week, and β is a vector of coefficients; *YEAR_t_* represents a set of indicator variables indicating calendar year to account for interannual variability, respectively, and γ is a vector of coefficients; *AT_t_*_,_*_t_*_–1_ is the average of the daily mean AT on day *t* and on the previous day [continuous, modeled as a spline function (*S*) with 6 degrees of freedom (df)]. We created several time series of AT for any combination of a forecast product and a lag period, for example, the time series of AT based on 1-day-ahead RDF consist of all ATs based on the RDF forecasts produced 1 day in advance; *T_t_* represents day of year (*T* = 1, 2… 365, spline function with df = 5) to account for seasonality; and η is the coefficient for the heat wave indicator variables. GAMs were fit using the “mgcv” R package (version 1.7–6) (Wood 2008) in the R statistical software (R Project for Statistical Computing; http://R-project.org). The parameters specifying the distribution of death counts were assigned by quasi-Poisson distributions to account for overdispersion.

We modeled AT–mortality associations and summarized heat effects by estimating the percentage difference in mortality associated with a given AT change. To facilitate comparisons across observations and forecasts, we used the GAMs described above to estimate the percentage difference in mortality on days with observed or predicted daily mean AT averaged over the same day and the previous day (AT_lag01_) of 25.3°C compared with 8.5°C for all data sets (observed and forecast). These temperature references represent the 90th and 50th percentiles of the observed daily mean AT distributions during 2002–2006, and they are consistent with the percentiles used by Anderson and Bell (2009). We considered a *p*-value < 0.05 to be statistically significant.

We conducted sensitivity analyses to examine whether estimated heat and heat wave effects differed when adjusted for O_3_ (on the same day), PM_10_ (on the previous day), or O_3_ and PM_10_ concentrations. We modeled both air pollutants using splines with 4 df, and selected the lag periods previously reported to have the strongest associations with mortality (Anderson and Bell 2010).

## Results

*Descriptive statistics*. Table 1 shows the RMSEs of daily average temperature, DPT, and AT metrics for each forecast product relative to the observed values. In general, for all products except MEX and ENS, RMSEs for all three weather parameters were lowest for predictions based on 1-day forecasts, and highest for predictions based on 3-day forecasts. The MAV forecast product produced the most accurate temperature and DPT predictions for all three forecast lengths, with 3-day predictions that were > 0.5°C closer to the observed values than predictions based on the other forecast products. The most accurate AT predictions were based on 1- and 2-day forecasts for MET, and 3-day forecasts for MAV. Not surprisingly, AT had the largest biases, followed by DPT, and temperature in general. We excluded the MEX and ENS products from further analysis because they had larger errors in 1-day forecasts compared with 2- and 3-day forecasts. The larger errors suggest some error characteristics in the 2- and 3-day forecast systems because forecasts typically have smaller errors with shorter lead time to issue forecasts. These systems were thus not appropriate for our application, focused on typical forecasts.

**Table 1:** Daily mean biases (average RMSEs) for TMP, DPT, and AT relative to observed values.

Forecast product	1-Day			2-Day			3-Day		
	TMP	DPT	AT	TMP	DPT	AT	TMP	DPT	AT
FWC	1.35	1.49	2.22	1.61	1.76	2.37	2.53	2.26	2.89
MAV	1.18	1.44	2.18	1.33	1.68	2.21	1.83	1.92	2.45
MET	1.20	1.65	2.07	1.41	1.81	2.15	2.79	2.12	3.14
MEX	2.33	2.26	2.80	1.66	1.78	2.45	1.91	2.04	2.66
ENS	2.21	2.18	2.69	1.66	1.76	2.44	2.02	2.08	2.72
RDF	1.49	1.58	2.11	1.58	1.75	2.30	2.61	2.33	3.31
Abbreviations: AT, apparent temperature; 1-day, forecast 1 day in advance; 2-day, forecast 2 days in advance; 3-day, forecast 3 days in advance; DPT, dew point temperature; TMP, forecast temperature.									

Table 2 compares numbers of heat wave days predicted by each forecast with the numbers of observed heat wave days, with heat waves defined as ≥ 2 consecutive days with daily mean AT ≥ 95th percentile. (The number of heat wave alerts or warnings that were issued by the local NWS office during the study period differed from the number of heat wave days defined according to the criteria above.) The number of predicted heat wave days varied from 11 days (based on the 3-day RDF forecast) to 22 days (based on 1- and 2-day FWC forecasts, and on the 2-day RDF forecast). For 1-day forecasts, which are more likely to be used to trigger a warning, all forecast products except RDF predicted more heat wave days than were observed. Patterns for 2- and 3-day forecasts were inconsistent. Most notably, 2-day RDF forecasts substantially overpredicted heat wave days (22 days compared with 14 observed during the study period), whereas 3-day RDF forecasts underpredicted heat wave days (11 days). Among all forecasts, 1-day RDF and 3-day MAV forecasts predicted the fewest false-positive heat wave days (5 days), compared with up to 13 false-positive days for other forecasts. Numbers of false-negative forecasts ranged from 2 (for the 2-day MAV forecast) to 11 days (for the 3-day RDF), with similar numbers (4 to 6 days) for all products based on 1-day forecasts. One-day forecasts correctly identified 8 to 10 of the 14 observed heat wave days during the study period.

**Table 2:** Heat wave days predicted by forecast products 1-, 2-, or 3-days in advance compared with heat wave days defined based on observed data, Detroit summers (1 May–30 September) 2002–2006.^*a*^

Observation and forecast products	1-Day				2-Day				3-Day			
	Total^*b*^	False positive^*c*^	False negative^*d*^	Correct	Total	False positive	False negative	Correct	Total	False positive	False negative	Correct
Observed^*e*^	14				14				14			
FWC	22	12	4	10	22	12	4	10	19	10	5	9
MAV	17	9	6	8	19	7	2	12	16	5	3	11
MET	21	11	4	10	20	10	4	10	20	13	7	7
RDF	13	5	6	8	22	11	3	11	11	8	11	3
Abbreviations: 1-day, forecast 1 day in advance; 2-day, forecast 2 days in advance; 3-day, forecast 3 days in advance. ^***a***^Heat wave days were defined as ≥ 2 days where apparent temperatures were ≥ 95th percentile values of apparent temperatures determined separately for each year. ^***b***^Total number of identified heat wave days. ^***c***^Days when the forecast product identifies heat waves that are defined as non–heat wave days in observed data. ^***d***^Observed heat wave days are incorrectly identified as non–heat wave days by forecasts. ^***e***^Weather observations in the Detroit Metropolitan Airport.												

**Figure 1:**
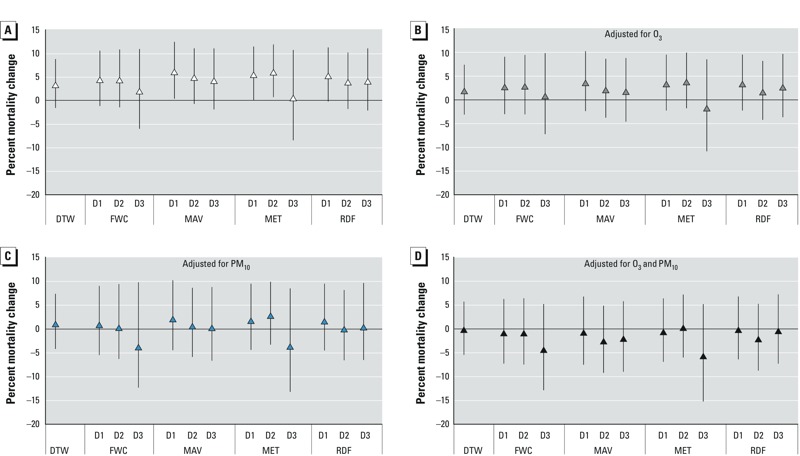
Estimated percentage difference in mortality associated with observed and forecast AT_lag01_ of 25.3°C compared with 8.5°C during the summertime (May–September) in Detroit, 2002–2006, with and without adjustment for air pollution. All models were adjusted for heat wave days, day of the week, day of the year, and calendar year. (*A*) No air pollution adjustment, (*B*) adjusted for same-day mean O_3_ concentration, (*C*) adjusted for mean PM_10_ concentration on the previous day, (*D*) adjusted for O_3_ and PM_10_ concentrations. Abbreviations: D1, forecast 1 day in advance; D2, forecast 2 days in advance; D3, forecast 3 days in advance; DTW, observed data.

*Heat effects*. The observations and most of the forecasts generated statistically nonsignificant excess relative risk estimates. The estimated percent increase in mortality associated with observed AT_lag01_ = 25.3°C versus 8.5°C was 3.5% (95% CI: –1.6, 8.8%) (Figure 1A). Associations between mortality and observed AT_lag01_, and most of the associations with predicted AT_lag01_, were positive but not statistically significant. Estimated associations with predicted AT_lag01_ varied among forecast products and time frames, with most overestimating the excess relative risk due to heat effects compared with associations based on observed AT_lag01_. For example, the estimated percent change in mortality based on AT_lag01_ predicted based on 2- and 3-day RDF forecasts was 4.0% (95% CI: –1.8, 10.2%) and 4.3% (95% CI: –2.1, 11.1%), respectively, whereas the estimate based on AT_lag01_ predicted from the 1-day MAV forecast was 6.2% (95% CI: 0.4, 12.5%). Interestingly, estimated increases in mortality associated with predicted AT_lag01_ based on 2- and 3-day RDF forecasts (produced by the local NWS office) were closer to associations with observed AT_lag01_ than associations with predicted AT_lag01_ based on other forecasts. Based on the width of the confidence intervals, relative risk estimates based on forecast AT_lag01_ were less precise than estimates based on observed AT_lag01_.

In general, patterns of estimated changes in mortality associated with observed or predicted AT_lag01_ = 25.3°C versus 8.5°C were similar after adjustment for ambient air pollution (Figure 1B–D). However, associations decreased in magnitude when adjusted for O_3_ only, PM_10_ only_,_ or both pollutants, and all point estimates became statistically nonsignificant.

**Figure 2:**
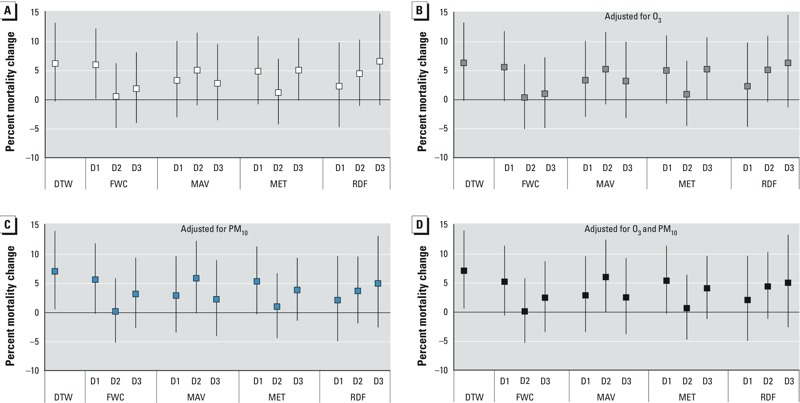
Estimated percentage difference in mortality associated with observed and forecast heat wave days compared with non–heat wave days during the summertime (May to September) in Detroit, 2002–2006, with and without adjustment for air pollution. All models were adjusted for AT_lag01_, day of the week, day of the year, and calendar year. (*A*) No air pollution adjustment, (*B*) adjusted for same-day mean O_3_ concentration, (*C*) adjusted for mean PM_10_ concentration on the previous day, (*D*) adjusted for O_3_ and PM_10_ concentrations. Abbreviations: D1, forecast 1 day in advance; D2, forecast 2 days in advance; D3, forecast 3 days in advance; DTW, observed data.

*Heat wave effects*. Associations between mortality and heat wave days versus non–heat wave days were statistically significant only for heat wave days predicted based on 1-day FWC forecasts (6.0%, 95% CI: 0.1, 12.2%) (Figure 2A). Associations with heat wave days classified based on observed AT were stronger (6.2% increase in mortality; 95% CI: –0.4, 13.2%) than associations with heat wave days predicted from forecasts, except for heat wave days predicted by the 3-day RDF forecast (6.6%, 95% CI: –1.0, 14.8%). Among all forecasts, associations with heat wave days predicted by the 1-day FWC forecast and the 3-day RDF forecast were the closest to the association with observed weather. In contrast to the heat effects discussed earlier, the uncertainty ranges (shown by width of confidence intervals) of the excess relative risk derived from all forecasts except for 1-day-ahead and 3-day-ahead RDF forecasts were smaller than those from the observations. Adjusting for O_3_, PM_10_, or both had little influence on associations between heat wave days and mortality (Figure 2B–D).

## Discussion

The present study addresses an epidemiologic question with potentially significant implications for the design of HHWS and the projection of future mortality risks attributable to heat and heat waves by conducting a case study in Detroit. The question was how does the performance of weather forecasts affect heat/heat wave–mortality associations and the likelihood of triggering an alert. Previous work has not examined this question systematically, which is an important omission given the increased trend of temperature and the growing frequency and severity of heat waves in a warm climate.

We explored six weather forecast products for Detroit derived from different algorithms that postprocess outputs from several numerical forecast models. Two of these, MEX and ENS, had error characteristics that suggested that they should not be used. We compared estimated associations of mortality with heat and heat wave days predicted using the four remaining forecast products to associations between mortality and observed heat and heat wave days. Our results suggest that, although the local operational forecast (i.e., RDF) was not always the most accurate in terms of biases compared with weather observations, it generally produced the estimates of heat and heat wave effects closer to associations with observed data than other forecast products. In addition, it produced far fewer false-positive calls, and similar numbers of false-negative calls, for heat waves. The estimated heat and heat waves effects varied with the forecast product and issuing time frames. The choice of forecast product could play a critical role in operating an HHWS more effectively.

Among the calculated weather metrics, AT was predicted with the largest bias, regardless of the forecast product used, followed by DPT and temperature. Not surprisingly, the accuracy in forecasting DPT was lower than that for temperature. Temperature is the more robustly forecast and spatially representative observation, and DPT is commonly thought to be more difficult to predict compared with temperature because it is largely affected by local land features such as lakes and rivers. AT is calculated from two directly forecasted variables (temperature and DPT), and thus it results in larger errors than either temperature or DPT. MEX and ENS had larger bias in temperature for a 1-day-ahead forecast than 2- and 3-day-ahead forecasts, possibly because large initialization errors in the forecasting systems were not much reduced at the 1-day time span.

The 1-day RDF forecast predicted the fewest false-positive heat wave days and an intermediate number of false-negative heat wave days compared with the other products. Although 1-day FWC and MET forecasts correctly predicted 2 more heat wave days than RDF, these products also predicted 6 or 7 more false-positive heat wave days. This finding suggests that the 1-day RDF forecast is an overall better product than others to use in order to issue heat alerts because the number of “wrong” alerts would be largely reduced when observed weather does not meet the heat alert criteria. This has important implications in risk communication because people do not trust heat warning systems if a system issues too many alerts. In addition, the 1-day RDF forecast had 8 correctly identified heat wave days compared with the observations that were similar to 8 to 10 days correctly predicted by using other forecast products. This suggests that using the RDF forecast product reduced the number of false-positive days with the cost of only a relatively lower number of correctly identified days. Although 2- and 3-day MAV forecasts predicted fewer false-negative heat wave days and correctly predicted more true-positive heat wave days than all other forecast products, local meteorologists pay more attention to the 1-day-ahead forecast and use all MOS products, as well as other forecast products, in their decision-making process for issuing a heat alert. Finally, this comparison across forecasts and weather observations highlighted the challenge in predicting weather extremes because all forecast models and postprocessing adjustments are designed for estimating the averages of temperature and other weather conditions (Lalaurette 2003).

Minor-to-significant differences of the estimated heat effects between forecasts and observations were observed across forecast products and time frames. Most of the forecast products overestimated associations between heat and mortality when compared with associations based on observed heat. The forecasts with the smallest biases may not necessarily result in the closest estimates of heat effects to those derived from the observations, and 1-day-ahead forecasts did not always result in estimated heat effects closer to those from observations than 2- or 3-day-ahead forecasts, suggesting that more information (e.g., bias propagation in forecast systems) is needed to better understand these relationships.

Compared with heat effects, associations between predicted heat wave days and mortality were smaller than associations with observed heat wave days and mortality. Forecast–mortality models captured heat wave effects less well than heat effects because forecast models perform worse in predicting extreme weather conditions. This suggests that we might expect more uncertainties in heat wave alerts issued by HHWSs compared with heat advisories. Heat wave alerts are more severe than heat advisories. In addition, although the forecasts made 1 day in advance are expected to be closer to the observations than other forecasts, their estimated associations with heat waves were not closer to the associations with the DTW observations than those estimates from other forecast periods, possibly because of the error propagation reason mentioned earlier. Overall, the comparison of heat/heat wave effects between observations and forecast demonstrated the importance of choice of weather forecast product in designing a HHWS.

Heat effects derived from both observations and forecasts were attenuated when models included O_3_, or PM_10_, or both, whereas estimated heat waves effects from forecasts and observations were similar. A recent national heat effects analysis for 107 U.S. communities reported that associations between heat and mortality slightly decreased with pollution adjustment in temperature-mortality models (daily mean temperature) (Anderson and Bell 2009). Unlike Anderson and Bell (2009), we estimated heat effects while controlling for heat waves in the same models. We acknowledge that, because of the lack of consensus on how to represent the joint and synergistic effects of heat/heat wave and air pollution on health, and practical challenges related to issuing alerts or warnings that take both heat wave and pollution into account, the practical application of the results adjusting for pollutants is currently limited.

The comparative analysis of weather forecasts presented here points out the challenge in issuing heat alerts based purely on numerical or statistical forecast models given that forecast quality varies with forecast products and issuing time frames. Heat alerts can activate public health interventions, and decisions on issuing them can depend not only on forecasts but also on NWS officials’ awareness of place-specific conditions (e.g., holidays, parades, fairs, major conferences in the area, health status of resident populations, and perhaps air quality forecasts in the future). Thus, local knowledge in both weather and population health, and cooperation among the meteorological, health, and social service sectors, not just forecasted conditions, are critical input in issuing heat warnings and alerts.

This study has a few limitations. First, our findings may be not generalizable to other cities, and further evaluation with data from more cities is needed. Second, some parameter specifications in our data analysis are subjective primarily because no consensus exists on the definition of heat waves and quantification of heat effects, for example, percentile-based heat wave definitions in summer months are different from the heat index used by NWS and may not capture all heat wave days. Duration was not considered in the analysis because of the small number of heat waves in the study period. Third, further work is needed to translate our findings into forecasters’ practice to support their decision making—and potentially improved triggers of heat alerts—because this is beyond the scope of this paper. Local forecasters make forecasts and heat alerts based on multiple forecast products as well as their judgment based on their local knowledge of historical weather and other factors mentioned above. Fourth, our findings on the impact of forecasts on heat–mortality associations are based on long time series of forecasted weather conditions, on average. In practice, forecasters look at forecasts a few days in advance and weather observations in just the previous days, so additional analyses, beyond the scope of this paper, would be required to evaluate the application of forecast data as heat alert triggers. One obstacle to this type of analysis is the difficulty in acquiring clean and reliable historical data sets showing which days were declared heat alerts or heat advisories by the NWS. We attempted to extract such information from archived NWS warnings, watches, and advisories, but we were stymied by the lack of uniformity in these documents. We believe that the development of improved heat alert triggers should account for forecast quality as well as many factors as discussed above. Choosing potentially improved triggers is a challenge because of the lack of consensus on the evaluation criteria as well as on the definitions of heat wave days. Potential criteria include which health outcomes to use as the “sentinel events” (e.g., mortality, hospital admissions, emergency department visits), robustness, false-negative and false-positive rates, effective communication about heat alerts to the public, and, perhaps, economic benefit–cost analysis. A final limitation of our analysis was that we used a mortality data set from which external causes had been previously excluded, thus impairing our ability to examine causes of death such as overdoses and intentional self-harm that may plausibly be linked to extremely hot weather. Future analyses could address questions regarding cause of death.

## Conclusions

Examining the impacts of weather forecasts on heat/heat wave–mortality associations and the performance of various forecast products in predicting heat waves is important for designing HHWSs and improving projection of heat-related health risks. Our analysis demonstrates the challenge in predicting health effects of weather extremes based on numerical and statistical forecast models. Forecasts showed higher associations between heat and mortality, and lower associations between heat waves and mortality, than observed weather. The impacts of weather forecast quality on mortality risk depended on forecast product and forecast time frames. Heat and heat wave effects derived from a local operational forecast product were generally closer to those calculated based on observations than those based on other forecast products. Our findings provide insights into issuing heat alerts and suggest that local knowledge on weather and population health are critical factors in HHWSs.
